# Integration of *c*-axis oriented Bi_3.15_Nd_0.85_Ti_2.95_Hf_0.05_O_12_/La_0.67_Sr_0.33_MnO_3_ ferromagnetic-ferroelectric composite film on Si substrate

**DOI:** 10.1038/s41598-017-11931-5

**Published:** 2017-09-12

**Authors:** Zongfan Duan, Ying Cui, Gaoyang Zhao, Xiaoguang Li, Biaolin Peng, Chunchun Han

**Affiliations:** 1 Shaanxi Key Laboratory of Electrical Materials and Infiltration Technology, School of Materials Science and Engineering, Xi’an University of Technology, Xi’an, 710048 China; 2 Department of Physics, University of Science and Technology of China, Hefei, 230026 China; 3 School of Physical Science and Technology, Guangxi University, Nanning, 530004 China; 4 CAS Center for Excellence for Nanoscience, National Center for Nanoscience and Technology, Beijing, 100190 China

## Abstract

A La_0.67_Sr_0.33_MnO_3_ (LSMO) ferromagnetic layer and a Nd^3+^/Hf^4+^ co-substituted Bi_4_Ti_3_O_12_ (Bi_3.15_Nd_0.85_Ti_3-x_Hf_x_O_12_ (BNTH_x_, x = 0, 0.025, 0.05, 0.1 and 0.15)) ferroelectric layer were successively deposited onto the (00 *l*)-oriented LaNiO_3_ (LNO) layer buffered (001) Si substrate via all chemical solution deposition (CSD) method. As a result, the BNTH_x_/LSMO ferromagnetic-ferroelectric composite films integrated on Si substrate exhibit high *c*-axis orientation. The Nd^3+^/Hf^4+^ co-substituted BNTH_x_ films have the lower leakage current and the better ferroelectric properties than the mono-substituted Bi_4_Ti_3_O_12_ (Bi_3.15_Nd_0.85_Ti_3_O_12_ and Bi_4_Ti_2.95_Hf_0.05_O_12_) films. In particular, the BNTH_0.05_/LSMO/LNO film has the lowest leakage current density of 2.5 × 10^−7^ A/cm^2^ at 200 kV/cm, and the highest remnant polarization (*P*r) of 27.3 μC/cm^2^. The BNTH_0.05_/LSMO/LNO composite film also exhibits the soft ferromagnetism characteristics with a high saturated magnetization of 258 emu/cm^3^ at 300 K, and the excellent magnetoelectric (ME) effect. The variations of ME voltage coefficient *α*_E_ values with DC bias magnetic field *H*_bias_ shows that the BNTH_0.05_/LSMO/LNO film has the high *α*_E_ value at near zero *H*_bias_. Moreover, at *H*_bias_ = 0 Oe, the *α*_E_ value gradually increases from zero with the increasing of the AC magnetic field frequency, and eventually reaches about 18.9 V/cm·Oe at 100 kHz, suggesting the existence of self-biased ME effect.

## Introduction

In multiferroic magnetoelectric (ME) materials, the coexistence of ferromagnetic and ferroelectric properties provides a possibility to obtain “magnetoelectric (ME) effect”, by which an induced electrical polarization and magnetization can be controlled by applying a magnetic and electric field, respectively. It would play important role in the novel multifunctional devices such as sensors, electric field-controlled magnetic data storage, actuators, spintronics, and microelectro-mechanical systems^1–4^. However, the ME effect of the available single-phase magnetoelectric materials is usually weak at low temperature. Recently it has attracted many researchers from the multiferroic ME field to develop new ferromagnetic-ferroelectric composite materials.

Moreover, to develop environmental friendly and new generation devices, considerable efforts have been made to prepare the lead-free ferroelectric materials and their corresponding ferromagnetic-ferroelectric composite films. Bi_4_Ti_3_O_12_ (BIT) as a bismuth-layered perovskite metal oxide material is one of the most popular materials owing to its low coercive field, low dielectric constant, high Curie temperature and high breakdown strength^5, 6^. However, some disadvantages such as the high leakage current, the domain pinning, and the poor fatigue endurance limit its further applications. Thankfully, the poor ferroelectric performance of BIT could be improved by an appropriate chemical substitution either in its A-site (Bi-site) or B-site (Ti-site) or both A and B-sites. Recently, the trivalent rare-earth ions such as Nd^3+^, La^3+^, Eu^3+^, Pr^3+^, Ce^3+^, Sm^3+^, Gd^3+^, have been used to partially substitute the A-site of BIT to enhance the chemical stability of oxygen vacancies in the perovskite block, and achieve a better fatigue endurance and reduce the leakage current density^7–13^. In particular, Chon *et al*. reported that the *c-*axis oriented Bi_3.15_Nd_0.85_Ti_3_O_12_ thin film deposited by a sol-gel method showed a switchable remnant polarization (*P*r) record of 51.5 μC/cm^2^ and a fatigue-free behavior^7^. Meanwhile, some large radius (Zr^4+^, Hf^4+^) or higher charge valence (Nb^5+^, W^6+^) ions have been used to partially substitute the B-site of BIT to enhance the ferroelectric properties by inducing the distortion of oxygen octahedra and reducing the space charge density^14–17^. For example, Zhu *et al*. reported that a (208)-oriented Hf-doped BIT was integrated with GaN using SrTiO_3_/TiO_2_ buffer layer through a pulsed laser deposition (PLD) method. Hf-doped BIT has a large *Pr* of 22.5 μC/cm^2^ and a very low leakage current density of 1.94 × 10^−7^ A/cm^2^ at the electric field of 200 kV/cm^15^. Furthermore, the co-substitution at A- and B-sites in BIT films has been proved to be the most effective to enhance their polarization and reduce their leakage current. For example, Nd^3+^/V^5+^, La^3+^/Mn^3+^, Pr^3+^/Nb^5+^, Nd^3+^/Zr^4+^ co-substituted BIT thin films have been proved to exhibit the better ferroelectric properties compared with the corresponding mono-substituted BIT counterparts^18–21^. Although Nd^3+^ or Hf^4+^ mono-substituted BIT materials has been well investigated, to our knowledge, the Nd^3+^/Hf^4+^ co-substituted BIT thin film has not been prepared and studied. It is necessary to investigate the ferroelectric properties of BIT thin film co-substituted by Nd^3+^ and Hf^4+^, and further prepare ferromagnetic-ferroelectric composite films using this new materials.

In the ferromagnetic-ferroelectric composite films, the ferromagnetic-ferroelectric layered composite film with a 2–2 layered type structure is most popular one since the leakage current can be significantly reduced in this kind of structure by isolating the low resistive ferromagnetic phases with some insulating ferroelectric phases^22^. Furthermore, it is relative easy to modulate or control the thickness, the lattice strain, the connectivity and the crystal orientation of ferromagnetic and ferroelectric phases. In the ferromagnetic-ferroelectric layered composite film, the crystal orientation significantly affects its ferromagnetic and ferroelectric properties, and its ME coupling behavior as well^22–24^. Recently, much work has been carried out to fabricate the oriented (or even epitaxial) ferromagnetic-ferroelectric layered film to obtain the better ME coupling performance. However, to obtain the preferred orientation, those films were usually deposited onto the expensive and small-sized single-crystal substrates such as LaAlO_3_, SrTiO_3_ and MgO^25–29^. It is well known that most microelectronic devices are integrated on a silicon substrate. To combine the ferromagnetic-ferroelectric composite film with other functional materials and develop new multi-functional devices, it would be very necessary for the ferromagnetic-ferroelectric layered film to orientedly grow onto the Si substrate^30^. For this strategy, some buffer layers must be used. LaNiO_3_ (LNO) is a very attractive candidate with a pseudocubic lattice parameter (0.384 nm), matching with most ferromagnetic and ferroelectric perovskite materials. The similarity in both the crystal structure and the lattice constants between the LNO layer and the ferroelectric (or ferromagnetic) layers would result in the better lattice matching and a favorable structure to improve the ferroelectric (or ferromagnetic) properties^31–33^. Nevertheless, to our knowledge, any work on the deposition of oriented lead-free ferromagnetic-ferroelectric composite films including the bismuth-layered perovskite phase on LNO buffered Si substrates are barely reported.

In this work, a (00 *l*)-oriented LNO buffer layer was firstly deposited onto the (001) Si substrate to promote the preferential orientation growth of the overlying La_0.67_Sr_0.33_MnO_3_ (LSMO) ferromagnetic and Bi_3.15_Nd_0.85_Ti_3−x_Hf_x_O_12_ (BNTH_x_, x = 0, 0.025, 0.05, 0.1 and 0.15) ferroelectric layers. And thus the *c*-axis oriented BNTH_x_/La_0.67_Sr_0.33_MnO_3_ ferromagnetic-ferroelectric composite films integrated onto the Si substrate were obtained. All layers were prepared by the low-cost and facile chemical solution deposition (CSD) method. The crystalline, microstructure, ferroelectric and ferromagnetic properties, and ME coupling effect of the as-prepared ferromagnetic-ferroelectric composite films were also discussed in detail.

## Results and Discussion

**Figure 1:**
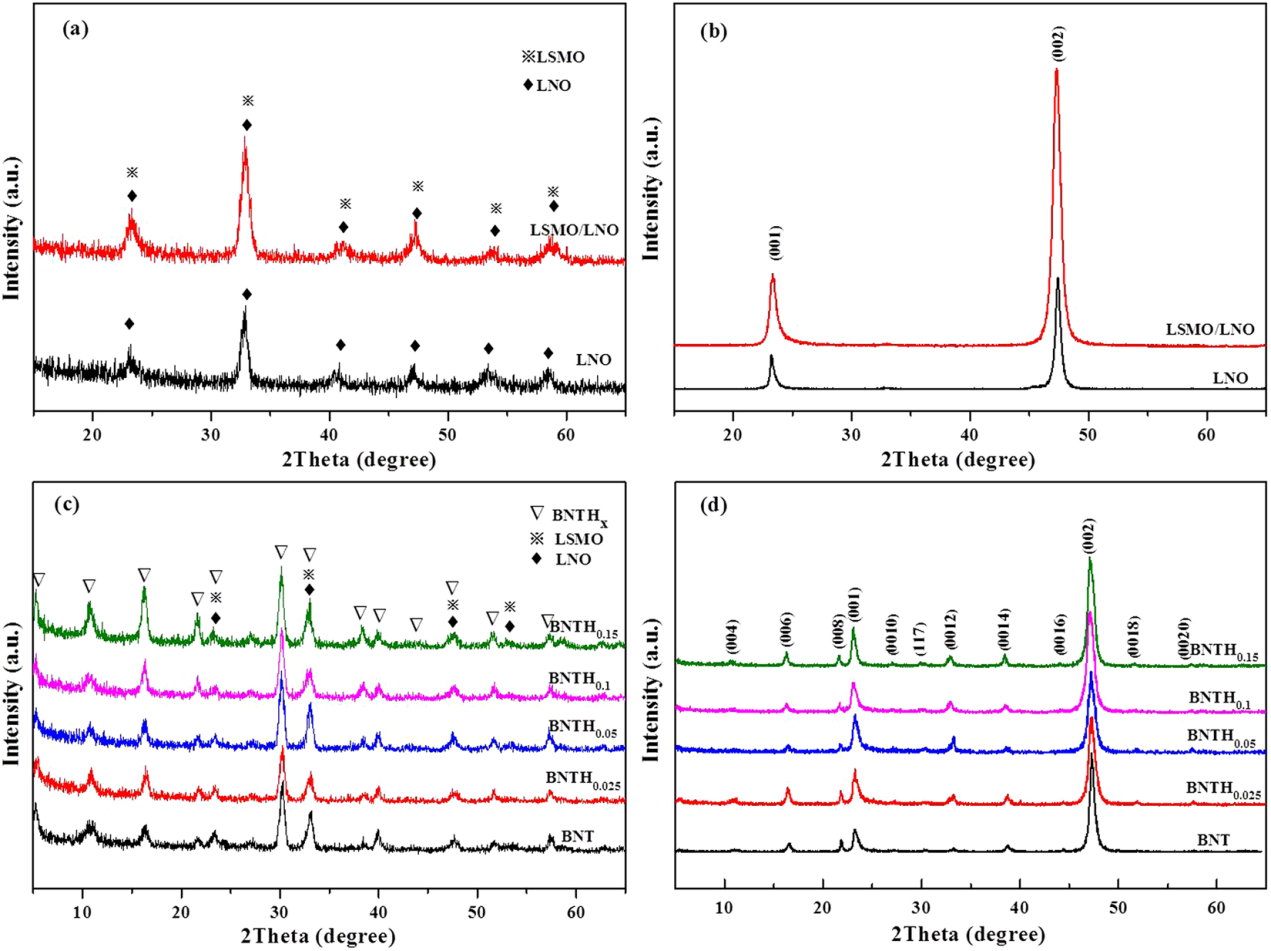
Low-angle (**a**,**c**) and θ-2θ (**b**,d) XRD patterns for LNO and LSMO/LNO films (**a**,**b**), and BNTH_x_/LSMO/LNO films (**c**,**d**).

The crystal structure and crystalline orientation of all films were characterized by low-angle and theta-2theta X-ray diffraction. The low-angle XRD patterns of LNO and LSMO/LNO are shown in Fig. 1(a). The diffraction peaks from the LNO and LSMO layers were satisfactorily indexed on the base of a cubic cell for LNO (according to the JCPDS standards, Card No. 33-0710), and a rhombohedral cell for LSMO (JCPDS 50-0308), respectively. The LNO and LSMO layers were well crystallized and free of impurity phases. The theta-2theta XRD patterns of LNO and LSMO/LNO films are shown in Fig. 1(b). In the LNO film, only the (00 *l*) (*l* = 1, 2) reflections exist, indicating the high *c*-axis orientation. In the LSMO/LNO film, the reflection of the LSMO layer completely overlapped that of the LNO layer. Because the pseudocubic LNO and LSMO phases have *a* lattice parameters of 0.384 and 0.387 nm respectively, the lattice match between LNO and LSMO was calculated to be more than 99%^34^. As a result, the LSMO layer grew on the LNO template in the same orientation. The orientation degree of LNO and LSMO/LNO films along the *c*-axis, which were calculated according to the Lotgering method, were as high as 98.84% and 99.54%, respectively. In general, the (00 *l*) type planes are the close-packed planes, the interfacial energy could be minimized by the formation of a highly *c*-axis oriented film. As a result, the LSMO layer would provide a template to grow a *c*-oriented BNTH_x_ layer. Figure 1(c) shows the low-angle XRD patterns of BNTH_x_(x = 0, 0.025, 0.05, 0.1 and 0.15)/LSMO/LNO films. It should be noted that the following BNTH_0_ would be represented by BNT. All the XRD patterns were identified and indexed according to the standard data of the Nd^3+^-substituted Bi_4_Ti_3_O_12_ (Bi_3.6_Nd_0.4_Ti_3_O_12_, JCPDS 36-1486). In addition, no any other peaks related to Nd and Hf, such as Nd_2_O_3_ and HfO_2_, were observed. This indicates that the bismuth-layered perovskite structures of BIT and Bi_3.15_Nd_0.85_Ti_3_O_12_ (BNT) were not destroyed, and Hf^4+^ was incorporated into BNT material in a way of substitution for Ti^4+^. The theta-2theta XRD patterns of BNTH_x_/LSMO/LNO films are shown in Fig. 1(d). Apart from (001) and (002) diffraction peaks of LSMO and LNO film layers, other (00 *l*) (*l* = 4, 6, 8, 10 and etc.) diffraction peaks also appear. This proves that the BNTH_x_ layers also grew along *c*-axis on the LSMO/LNO film. In addition, the calculated *c*-axis orientation degrees of all BNTH_x_/LSMO/LNO films are more than 99.0%.

**Figure 2:**
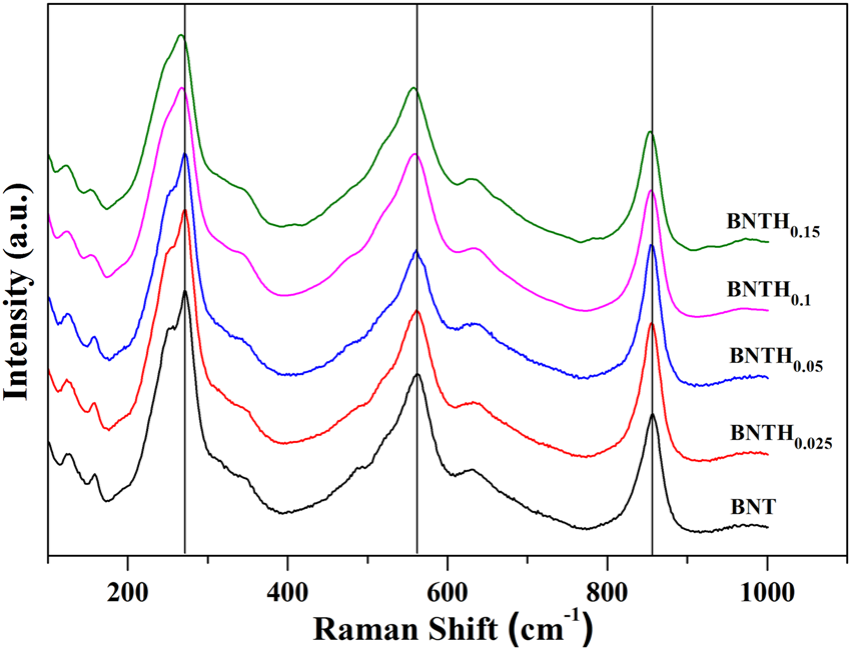
The Raman spectra of BNTH_x_ powders.

The Raman spectra of BNTH_x_ (x = 0, 0.025, 0.05, 0.1 and 0.15) powders at room temperature are shown in Fig. 2. The modes above 200 cm^−1^ are assigned with the TiO_6_ octahedra^35^, the ones at ~271 cm^−1^ correspond to the torsional bending of TiO_6_ octahedra, the ones at ~855 cm^−1^ relate to the stretching of the O-Ti octahedral chain between two (Bi_2_O_2_)^2+^ layers, and the ones at ~562 cm^−1^ arise from a combination of stretching and bending of the TiO_6_ octahedra^36–38^. Compared with pure BNT, the low frequency shift was observed in BNTH_x_ samples in those three modes when the content of Hf^4+^ substitution increased. This is because the substitution of heavy Hf^4+^ for Ti^4+^ has a great effect on the vibration modes of the TiO_6_ octahedra^39, 40^. So it could be concluded that the heavier Hf^4+^ entered into the lattice of BNT by substituting the lighter Ti^4+^ into B-sites. Because the ionic radius of Hf^4+^ (0.071 nm) was about 16% larger than that of Ti^4+^ (0.061 nm), the substitution of Ti^4+^ by Hf^4+^ would lead to the octahedral distortion in BNTH_x_^15, 41^.

**Figure 3:**
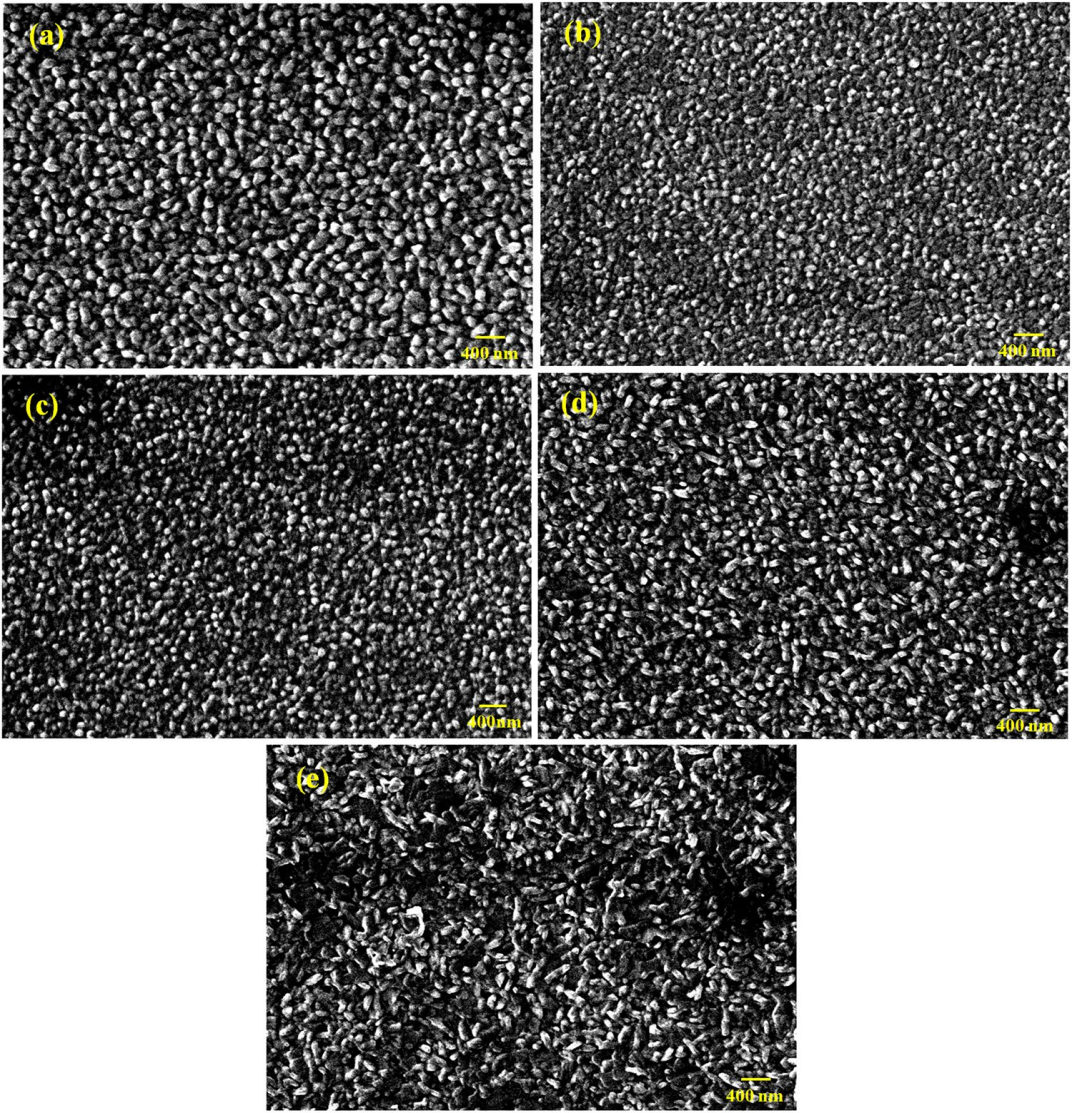
Surface SEM images of the BNTH_x_ layers deposited on LSMO/LNO films: BNT (**a**), BNTH_0.025_ (**b**), BNTH_0.05_ (**c**), BNTH_0.1_ (**d**), BNTH_0.15_ (**e**).

The surface morphologies of BNTH_x_ layers deposited onto the LSMO/LNO films are shown in Fig. 3. The BNT, BNTH_0.025_ and BNTH_0.05_ films all have smooth and dense surfaces (Fig. 3a–c). Three kinds of films are characterized by well-shaped spherical grains and a relatively narrow grain size distribution. No any pinholes are observed. The grain sizes of the BNTH_0.025_ and BNTH_0.05_ films are relatively smaller than that of the BNT film. It is possible due to the lattice distortion induced by the Hf^4+^ substitution, which likely slows down the growth rate of grains. In BNTH_0.10_ and BNTH_0.15_ films (Fig. 3d and e), the grains with both elongated and plate-like shapes are observed. The number of the plate-like grains in BNTH_0.15_ film is obviously much more than that of elongated grains, which is different from BNTH_0.10_ film. The microstructural difference among the BNTH_x_ films is believed to be related to the Hf^4+^-substitution content. Obviously, the lattice distortion induced by Hf^4+^-substitution at the B-site of BNT results in the change of grain morphology. With the increase of Hf^4+^-substitution content, as a whole, the morphologies of BNTH_x_ grains have an evolution trend from spherical, elongated to plate-like shape.

**Figure 4:**
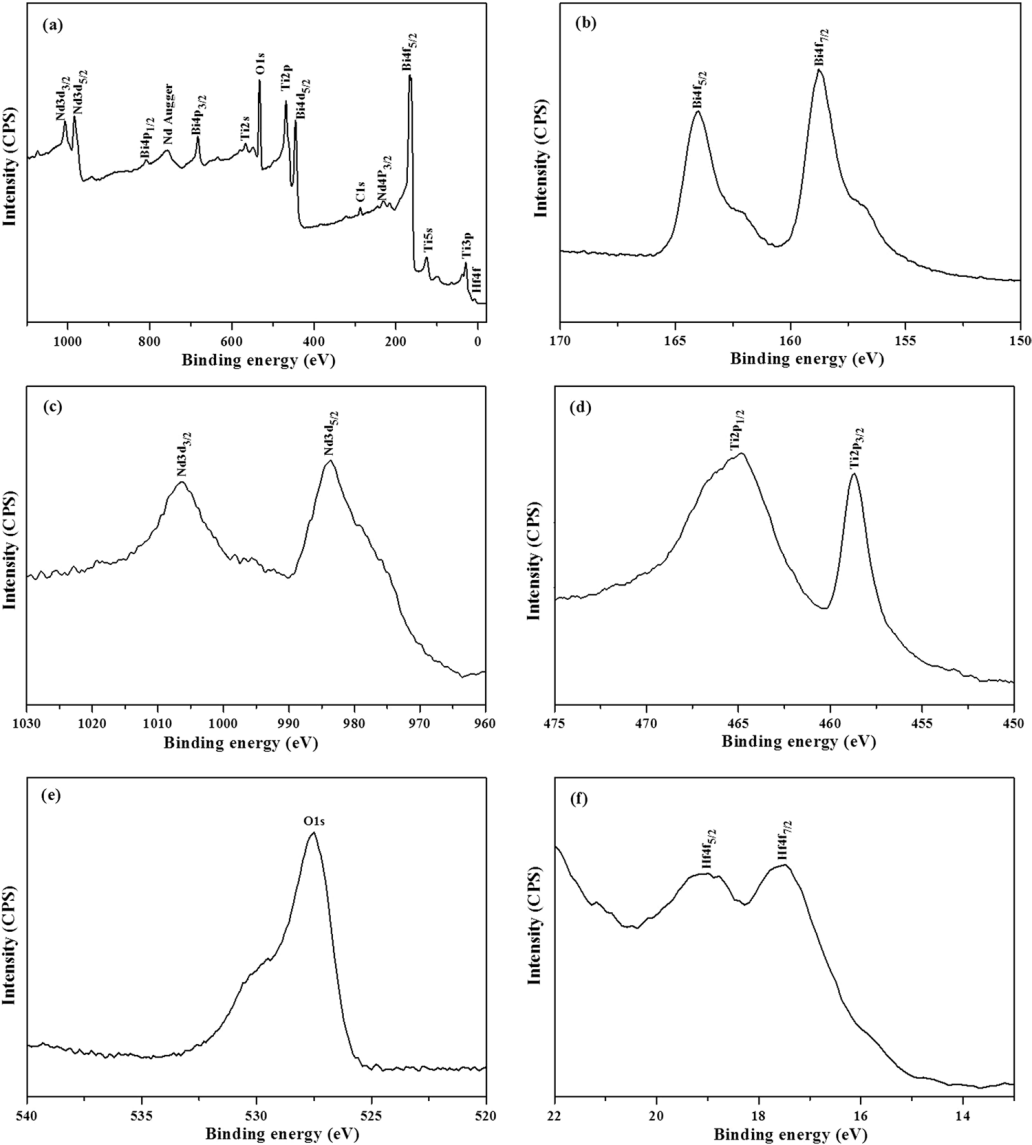
The XPS spectra of BNTH_0.05_ film: wide-scan spectrum (**a**), the narrow scan spectrum of Bi4f (**b**), Nd3d (**c**), Ti2p (d), O1s (**e**) and Hf4f (**f**).

In order to elucidate the element composition and the chemical state of the BNTH_x_ films, the BNTH_0.05_ film as a representative sample was characterized by the X-ray photoelectron spectroscopy (XPS), as shown in Fig. 4. The peak positions of different atoms were calibrated by internally referencing the adventitious carbon at a binding energy of 284.6 eV. As evident in the Fig. 4(a), the primary features are dominated by the peaks, which are attributed to Bi4f, Bi4d, Nd3d, Ti2p, Ti3p, O1s and Hf4f, etc. Except for the surface adventitious carbon, there is no any indication for the presence of any impurity atoms. According to the narrow scan of the Bi4f (Fig. 4b), the Bi4f spin-orbit has doublet peaks, located at 164.1 eV (Bi4f_5/2_) and 158.7 (Bi4f_7/2_). These bonding energy levels are consistent with the data of Bi_2_O_3_ powder^42^. This indicates that Bi of the BNTH_0.05_ phase exists in a form of Bi^3+^. The peaks around 1006.3 and 984.1 eV are attributed to the binding energies of Nd3d_1/2_ and Nd3d_3/2_with trivalent chemical state, respectively (Fig. 4c). They were consistent with the data of Nd_2_O_3_^43^. Figure 4(d) illustrates the fine peaks attributed to Ti2p core levels. The Ti2p spectrum was complicated due to the multiple splitting (Ti^4+^ and Ti^3+^). From the binding energy of 464.8 eV of Ti2p_1/2_ and 458.7 eV of Ti2p_3/2_, it could be inferred that the oxidation state of Ti ion was quite likely 4^+^ in the deposited BNTH_0.05_ layer^44^. The narrow spectrum of O1s is shown in Fig. 4(e), the peak centered at 527.6 eV is mainly assigned to the oxygen in the BNTH_0.05_ lattice^45^. As shown in Fig. 4(f), the Hf4f spin-orbit has doublet peaks located at 19.1 eV (Hf4f_5/2_) and 17.5 (Hf4f_7/2_). It is assigned to Hf-O bonding. The oxidation state of Hf ion is quite likely 4^+^^46^.

**Figure 5:**
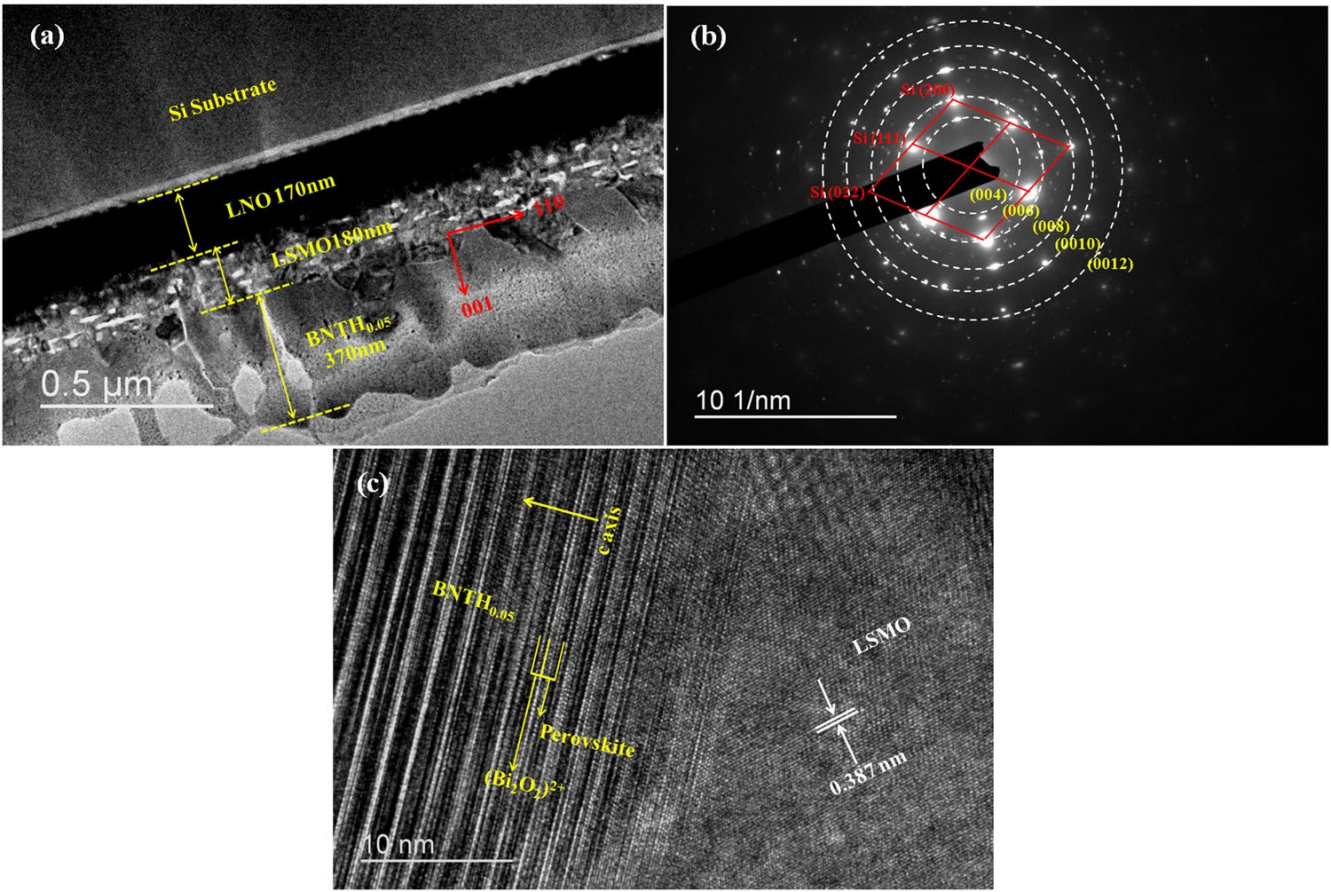
The cross-sectional (**a**) and SAED pattern (**b**) of the BNTH_0.05_/LSMO/LNO composite film, and HRTEM image (**c**) of BNTH_0.05_/LSMO interface.

The high-resolution transmission electron microscopy (HRTEM) analysis was performed on the BNTH_0.05_/LSMO/LNO composite film to further investigate the preferential orientation of individual layers with respect to the Si substrate. The TEM cross-sectional image of the BNTH_0.05_/LSMO/LNO heterostructure film is shown in Fig. 5(a). Each layer was clearly observed. The thicknesses of LNO, LSMO and BNTH_0.05_ layers are 170, 180 and 370 nm, respectively. A selected area electron diffraction (SAED) of the BNTH_0.05_/LSMO/LNO film is shown in Fig. 5(b). The sample is a polycrystalline film with a continuous and clear diffraction rings corresponding to (004), (006), (008), (0010) and (0012) crystal planes. It is consistent with the XRD results (Fig. 1d), and further confirms that the composite film exhibits a *c*-axis orientation. The high-resolution TEM image of the interface between BNTH_0.05_ layer and LSMO layer is shown in the Fig. 5(c). The measured interplanar spacing of the LSMO film is about 0.387 nm, which is consistent with the lattice parameters of the pseudocubic structure of LSMO (JCPDS 50-0308). The BNTH_0.05_ layer has a relatively uniform contrast without any indication of the grain boundaries in the view area. The fringes with different contrasts appear in a regular period. The perovskite unit of the BNTH_0.05_ structure corresponds to a slab of relatively low contrast, and the (Bi_2_O_2_)^2+^ unit appears as a line of white spots fringed with the dark contrast^47^.

**Figure 6:**
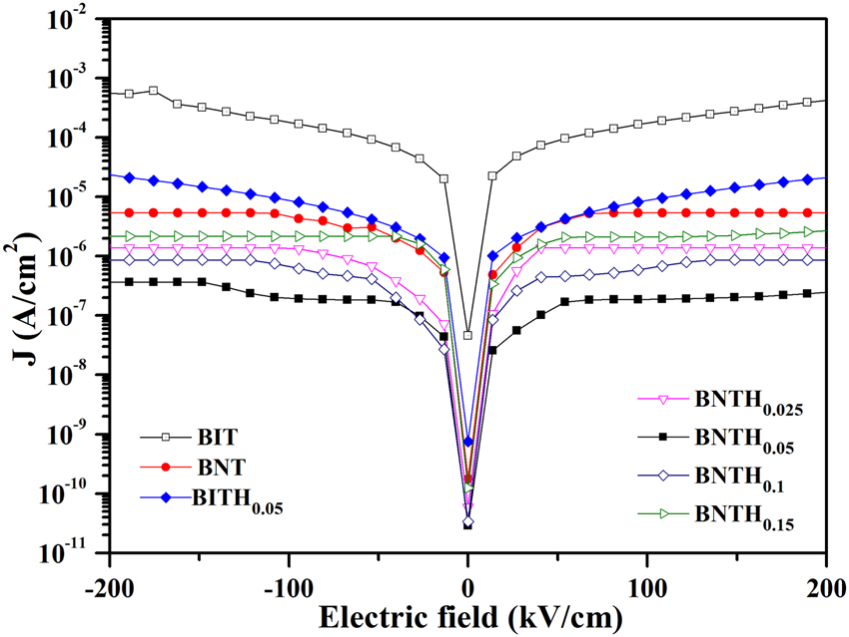
The leakage current density as a function of electric field in BIT, BNT, NITH_0.05_ and BNTH_x_ (x = 0.025, 0.05, 0.1 and 0.15) films.

Since LNO is a conductive metal oxide, it can be used as the bottom electrode material to measure the leakage current density, the polarization-electric field (P-E) hysteresis loop and the ME coupling effect. In order to investigate the effect of Nd^3+^ and Hf^4+^ substitution on the leakage current and the ferroelectric properties of BNTH_x_ layers deposited onto LSMO/LNO films, the electric properties of pure BIT and Hf^4+^-substituted BIT (Bi_4_Ti_2.95_Hf_0.05_O_12_, noted as BITH_0.05_) layers as counterparts were also determined. The leakage current density (J) was plotted against the applied electric field (E) for all the films. As shown in Fig. 6, the leakage current density of all the films increases gradually with the applied electric field. There was no significant difference in the leakage behavior when the electric field was reversed. In the pure BIT film, since Bi is very easy to volatilize during the heat treatment, it would create oxygen vacancies. These vacancies may act as the trap sites to deteriorate the leakage properties of the film^7^. As a result, the leakage current density in the BIT film was as high as 4.3 × 10^−4^ A/cm^2^ at the maximum electric field of 200 kV/cm. Fortunately, the lanthanide Nd^3+^ as a substitution element has the chemical property of the non-volatile at the high temperature. So the partly substitution of Bi^3+^ would enhance the stability of perovskite-like structure and reduce the oxygen vacancy concentration of the film, lowering the leakage current density^7, 48^. As we expected, the leakage current density of BNT film was reduced by two orders of magnitude (5.4 × 10^−6^ A/cm^2^) compared with that of BIT film at 200 kV/cm. The BITH_0.05_ film also shows a decreased leakage current density of 2.0 × 10^−5^ A/cm^2^ at 200 kV/cm. The leakage current was reduced by the substitution of Hf^4+^ at B-site (Ti-site) of BIT. This suggests that the insulation properties were improved. It is well known that the nature of Ti ion has variable valance, and its valance state can be often changed from Ti^4+^ to Ti^3+^ through the following reaction:

1 $$2{{\rm{Ti}}}^{4+}+{{\rm{e}}}^{-}\to 2{{\rm{Ti}}}^{3+}+V\ddot{O}$$

The electron, which was captured by the Ti^4+^ to become Ti^3+^, would not be very tightly bound to that ion. A little thermal agitation can easily liberate this kind of electron. The system can behave as n-type semiconductor, increasing the conductivity of the films and bringing about the leakage current^19^. The ionic radius of Hf^4+^ is larger than that of Ti^4+^. The substitution of Ti^4+^ by Hf^4+^ can not only block the passage of the two adjacent Ti ions, but increase the ion transition distance. As a result, in the BITH_0.05_ film, the conduction induced by the electronic transition between Ti^4+^and Ti^3+^ was reduced, the leakage current decreased obviously. The leakage current densities of BNTH_0.025_, BNTH_0.05_, BNTH_0.10_ and BNTH_0.15_ films were 1.4 × 10^−6^, 2.5 × 10^−7^, 8.6 × 10^−7^ and 2.6 × 10^−6^A/cm^2^ at 200 kV/cm, respectively. Those leakage current values were all lower than those of mono-substituted BIT (BNT and BITH_0.05_) films. In another words, owing to the joint contribution from Nd^3+^ (A-site) and Hf^4+^ (B-site) substitutions, the Nd^3+^/Hf^4+^ co-substituted BIT films have the lower leakage current performance. The substitution content had a great effect on the properties of substituted BIT films, and the excessive ion substitution might result in the deterioration of electric properties of the BIT films. It has been observed by many groups^19, 49^. The leakage current density of the as-prepared BNTH_x_ films decreased firstly, and increased afterward with the increasing of the content of Hf^4+^ substitution. The lowest leakage current density of BNTH_0.05_ film was 2.5 × 10^−7^ A/cm^2^ at 200 kV/cm. In the previous study, Wang *et al*. studied the dependence of the electrical properties of Bi_3.15_Nd_0.85_Ti_3−*x*_Zr_*x*_O_12_ thin films with a highly preferred (117) orientation on the content of Zr^4+^ substitution. They found that the variation of the leakage current density with Zr^4+^ substitution content were attributed to the orientation degree of the films along the *a*-axis^21^. However, in this work, all BNTH_x_ films exhibited the *c*-axis orientation, and the calculated orientation degrees were more than 99.0% (Fig. 1). So the effect of the orientation degree on the leakage current of the as-prepared BNTH_x_ films was negligible. It is well known that the microstructures, such as grain shape and size, density, and smoothness of films also have a significant effect on their electric properties. The increase of the leakage current densities in BNTH_0.10_ and BNTH_0.15_ films is likely ascribed to the evolution of grains shape and the accompanying deterioration of density and roughness of the films. Furthermore, the impurity phase derived from the excessive Hf^4+^ might segregate at the boundaries acting as the defect at domain walls, and affect the leakage current^19, 49^.

**Figure 7:**
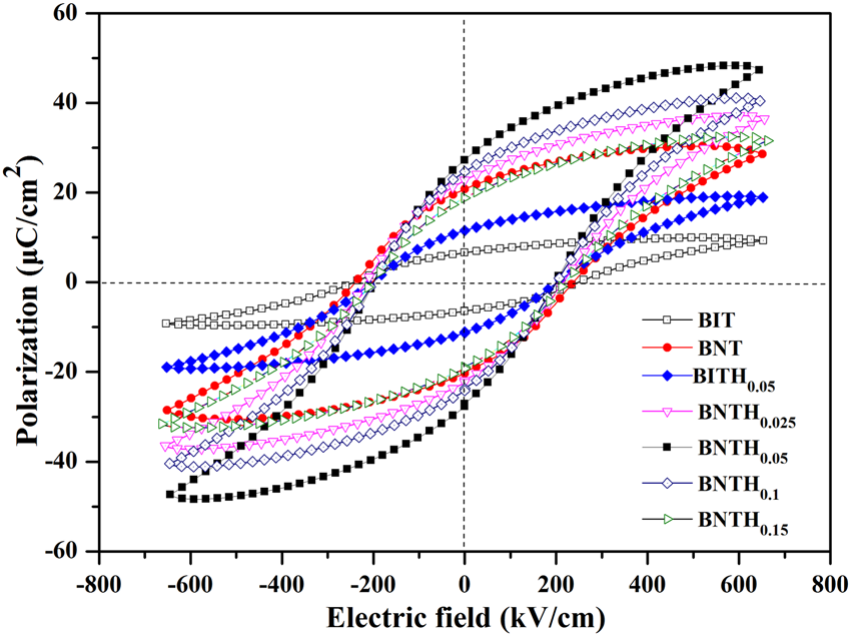
Room-temperature ferroelectric hysteresis loops of BIT, BNT, NITH_0.05_ and BNTH_x_ (x = 0.025, 0.05, 0.1 and 0.15) films.

Figure 7 shows the room-temperature polarization-electric field (P-E) hysteresis loops of the BIT, BITH_0.05_ and BNTH_x_ films, which were measured by the applied electric field up to 650 kV/cm at a frequency of 100 Hz. It can be seen that all samples exhibit well saturated hysteresis loops. The BIT film shows a polarization loop with the saturation polarization (*P*s) of 9.3 μC/cm^2^, *P*r of 6.7 μC/cm^2^ and coercive field (*E*c) of 248 kV/cm. The ferroelectric performance of the BIT film is not ideal owning to the high *c*-axis orientation. It has been previously proved that the polarization direction of BIT is 4.5° off the base plane in its cell structure, and the BIT thin film with a strong *c*-axis orientation is not desirable to have the high polarization^6^. However, the *c*-axis orientation is benefit for the BNT film to obtain the excellent ferroelectric properties. Chon *et al*. have proved that the high polarization in BNT film is attributed to TiO_6_ octahedron unit adjacent to the interleaving Bi_2_O_2_ layer, rather than the TiO_6_ unit of the inner central octahedron layer. And thus the direction of polarization is along the *c*-axis^7, 50^. As we expected, the *P*r value of the as-prepared BNT film with the high *c*-axis orientation was 20.8 μC/cm^2^. It was higher than that of BIT film, and comparable with those similar materials deposited onto other substrates^50, 51^. However, the highest *P*r record of 51.5 μC/cm^2^ was achieved in the *c*-axis oriented BNT thin film, which was deposited onto Pt/TiO_2_/SiO_2_/Si via a CSD process^7^. The difference of *P*r might derive from many factors such as substrates, electrodes, crystallinity, morphology, and so on. Compared with BIT film, the BTH_0.05_ film also has the better ferroelectric properties, *P*s of 19.2 μC/cm^2^, *P*r of 11.5 μC/cm^2^ and *E*c of 202 kV/cm. The ferroelectric properties of the BIT film could also be improved by the B-site (Ti-site) substitution of Hf^4+^. It was attributed to the distortion of oxygen octahedra and the decrease of the space charge density induced by B-site Hf^4+^ substitution^15^. BNTH_0.025_, BNTH_0.05_, BNTH_0.10_ and BNTH_0.15_ films all show well saturated loops due to their low leakage current properties. The *P*r values of the BNTH_0.025_, BNTH_0.05_, BNTH_0.10_ and BNTH_0.15_ films were 22.7, 27.3, 24.6, and 18.8 μC/cm^2^, respectively. The *P*r values of both BNTH_0.25_ and BNTH_0.05_ films were higher than those of BIT, BNT and BITH_0.05_ films. It can be concluded that the ferroelectric properties of the BIT film could be improved by the moderate Nd^3+^/Hf^4+^ co-substitution. The *E*c values of the BNTH_0.025_, BNTH_0.05_, BNTH_0.10_ and BNTH_0.15_ films were 214, 198, 200, and 220 kV/cm, respectively. The *E*c value of the ferroelectric materials has something do with the pinning effects of space charge and leakage current. For BNTH_x_ films, the dependence of *E*c on the Hf^4+^ substitution content was agreed well with the dependence of the leakage current density on the Hf^4+^ substitution content. The BNTH_0.05_ film with the lowest leakage current density (Fig. 6) has the smallest *E*c value. On the whole, the BNTH_0.05_ film has the highest *P*s of 48.1 μC/cm^2^ and the highest *P*r of 27.3 μC/cm^2^, and the lowest *E*c of 198 kV/cm. Therefore, the BNTH_0.05_ film would have the potential application in the functional devices based on ferroelectric films.

**Figure 8:**
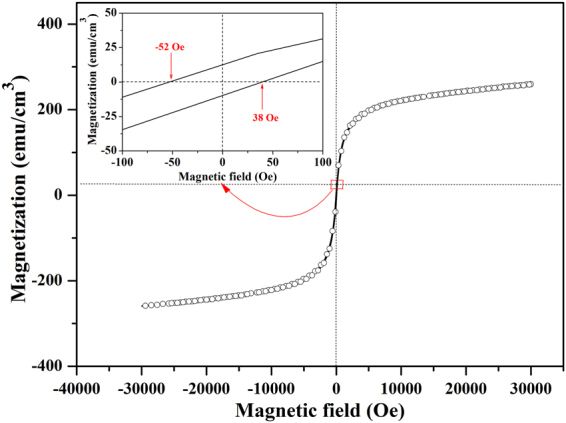
Magnetization hysteresis loop of the BNTH_0.05_/LSMO/LNO film.

Considering the best ferroelectric properties of the BNTH_0.05_/LSMO/LNO film, its ferromagnetic behavior and ME coupling behavior were well investigated. The magnetic hysteresis loop of the BNTH_0.05_/LSMO/LNO film was measured at 300 K, and the plane of the film was fixed to be perpendicular to the magnetic field. As evident in the enlarge view at near zero magnetic field of Fig. 8, the negative and positive coercive field values are 52 and 38 Oe, respectively. The absolute value of negative coercive field is higher than that of the positive coercive field, suggesting the existence of the exchange-bias effect (EBE) originated from LSMO/LNO interface^52^. Both coercive field values are low. This confirms that the composite film has the typical soft ferromagnetism characteristics because of the soft magnetization of LSMO phase. The film had a good ferromagnetic performance, and its saturated magnetization (Ms) value is 258 emu/cm^3^, which is comparable with previously reported values^23, 53^.

**Figure 9:**
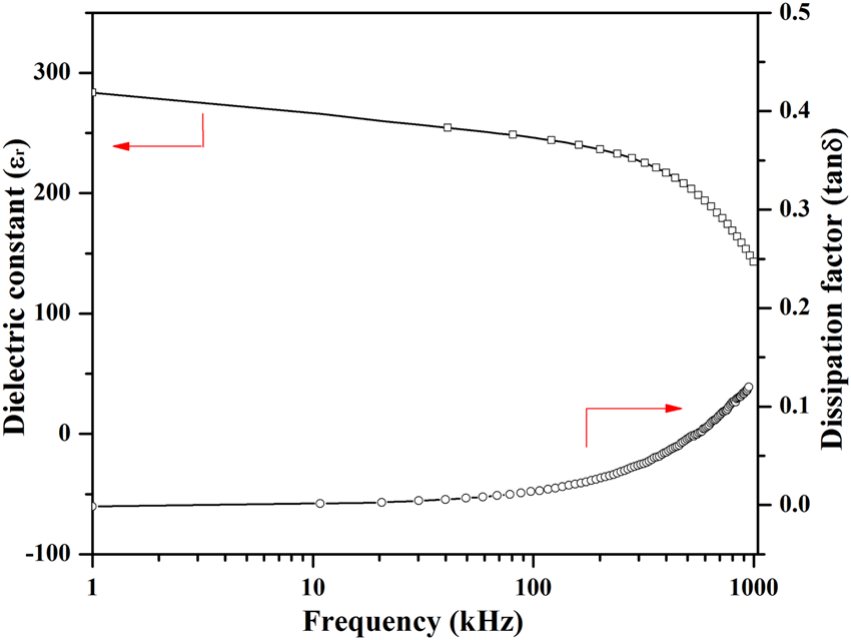
Variation of dielectric constant and dissipation factor as a function of frequency for the BNTH_0.05_/LSMO/LNO film.

The dielectric properties of BNTH_0.05_/LSMO/LNO film were evaluated by the dielectric constant (ε_r_) and the dissipation factor (tanδ). Figure 9 shows the variation of the dielectric constant and the dissipation factor as a function of the frequency for the film measured at room temperature. It is obvious that the dielectric constant slowly decreases with the increase of the frequency. There are almost no sudden changes of ε_r_ in the frequency range from 1 kHz to 100 kHz. However, its dielectric constant quickly drops in the frequency range from 100 kHz to 1000 kHz. The dissipation factor is very moderate and shows an opposite tendency.

**Figure 10:**
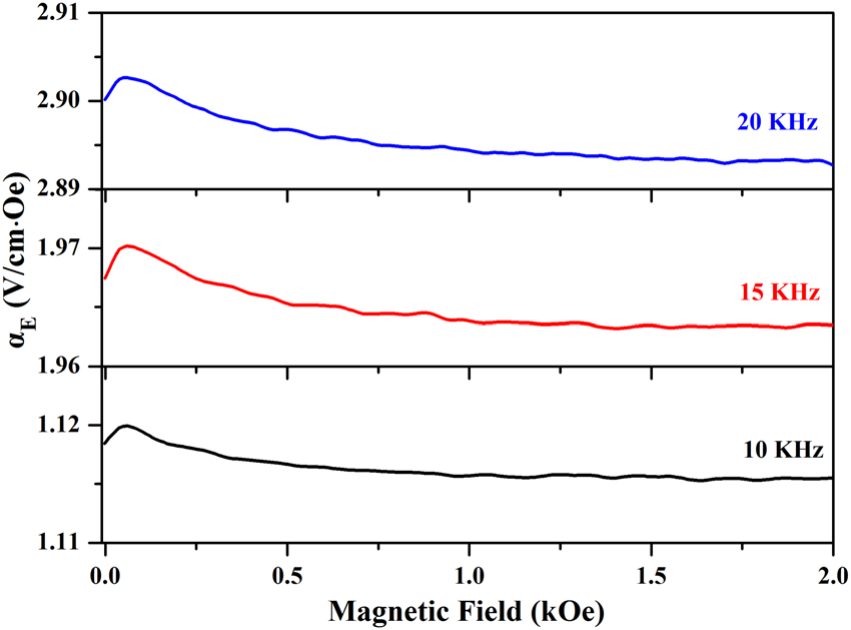
Variations of *α*_E_ with *H*_bias_ at various magnetic frequencies for the BNTH_0.05_/LSMO/LNO film.

**Figure 11:**
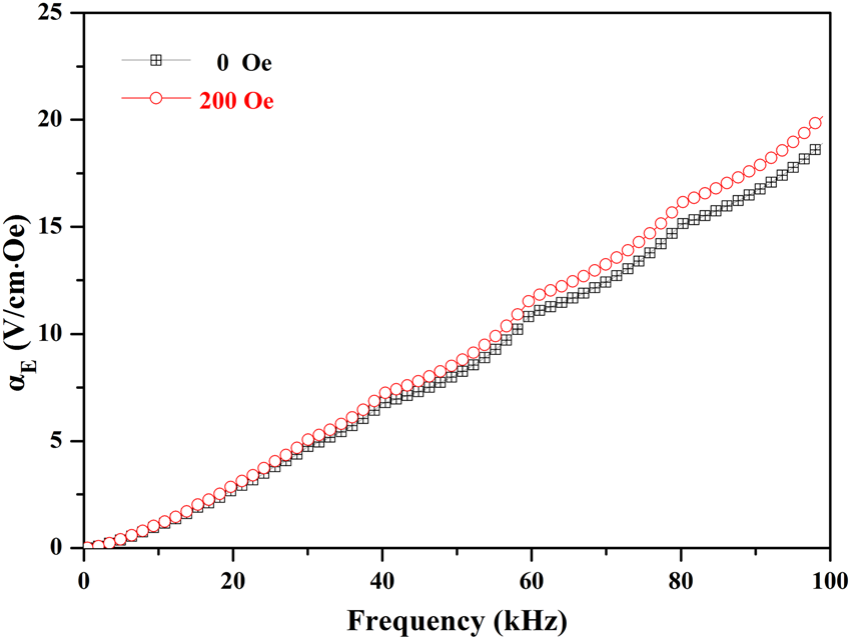
The frequency dependence of *α*_E_ for the BNTH_0.05_/LSMO/LNO film.

The ME effect is a product behavior derived from the coupling between the piezoelectric property of ferroelectric phase and the magnetostrictive effect of ferromagnetic phase, that is to say, an induced electrical polarization can be controlled by applying a magnetic field, contrarily an induced magnetization can be regulated by an electric field. For the as-prepared BNTH_0.05_/LSMO/LNO ferromagnetic-ferroelectric composite film, the ferromagnetic and ferroelectric phases have the outstanding single phase properties. It is beneficial to obtain the excellent ME effect. The ME effect could be expressed by the ME voltage coefficient, *α*_E_, which can be defined as:

2 $${\alpha }_{E}=\frac{\delta E}{\delta H}=\frac{\delta V}{t\times \delta H}=\frac{{V}_{out}}{(t\times {H}_{ac})}$$

where *V*_*out*_ is the induced voltage, *t* the thickness of the film and *H*_ac_ the alternating current (AC) magnetic field^54^. The dynamic ME signals of the BNTH_0.05_/LSMO/LNO film were measured by a lock-in amplifier at a small *H*_ac_ = 0.5 Oe under different magnetic frequencies of 10, 15 and 20 kHz. *H*_ac_ was driven by a pair of Helmholtz coil, and imposed with a direct current (DC) bias magnetic field *H*_bias_ (0–2 kOe). The direction of the magnetic field was perpendicular to the film plane. Figure 10 shows the *α*_E_ variation of the BNTH_0.05_/LSMO/LNO film with *H*_bias_ at different magnetic frequencies of 10, 15 and 20 kHz. At 10 kHz, the *α*_E_ value initially increases with *H*_bias_ increasing until reaching a peak, and then decreases to a nearly constant level with further increasing of *H*_bias_. The maximum *α*_E_ value of 1.12 V/cm·Oe was obtained at *H*_bias_ = 61.4 Oe. The ME behaviors of the film at 15 and 20 kHz showed the similar trends, and their corresponding maximum *α*_E_ value were 1.97 and 2.90 V/cm·Oe, respectively. Viewed as a whole, the *α*_E_ value at the near zero *H*_bias_ magnetic field was very large, but its variation with *H*_bias_ was modest. The similar behaviors were already observed by many groups^28, 29, 55–60^. It is well known that, in ferromagnetic-ferroelectric composite films, the ME coupling arises from the AC field initiated dynamic Joule magnetostriction caused by domain wall motion and rotation. The *H*_bias_ dependence of *α*_E_ is related to the magnetostriction of the ferromagnetic phase, and the high magnetostriction will result in the better dynamic magneto-elastic coupling, and producing a large ME effect^55, 61^. The BNTH_0.05_/LSMO/LNO film exhibited a strong ME effect in the near zero magnetic field. It was attributed to the larger magnetostriction of the LSMO phase. As discussed in ferromagnetism characterization (Fig. 8), the BNTH_0.05_/LSMO/LNO film has a low coercive field. This indicates that the LSMO phase had an easy-magnetization characteristic that was beneficial for the magnetic domain wall motion and rotation, leading to a larger magnetostriction even under a very low magnetic field. As a result, the large *α*_E_ values in the as-prepared BNTH_0.05_/LSMO/LNO film were obtained in the near zero magnetic field^55^. However, at *H*_bias_ = 0 Oe, *α*_E_ of the film is not equal to zero. This indicates that there may be self-biased magnetoelectric effect in the BNTH_0.05_/LSMO/LNO film. In order to explain this phenomenon, the *α*_E_ value of the composite film depended on the frequency of *H*_ac_ at *H*_bias_ = 0 Oe was investigated. Figure 11 illustrates the ME response of the film at *H*_ac_ = 0.5 Oe with the frequency from 0 to 100 kHz under *H*_bias_ = 0 Oe. Interestingly, the ME value of the composite film strongly depends on the frequency of *H*_ac_. The *α*_E_ value gradually increases from zero with the increasing of the AC magnetic field frequency, and eventually reaches about 18.9 V/cm·Oe at 100 kHz. The large *α*_E_ values were obtained in the absence of *H*_bias_, suggesting the existence of self-biased magnetoelectric effect. It is necessary to illustrate that the self-biased magnetoelectric effect was previously observed in three-phase metal-magnetoelectric ceramic laminate composites when the laminates were operated in bending mode and consisted of dissimilar or graded magnetic materials that resulted in built-in magnetic bias^62–65^. In present work, the emergence of the self-biased magnetoelectric effect in the BNTH_0.05_/LSMO/LNO film might be attributed to two causes. Firstly, as discussed in magnetization characterization (Fig. 8), there is an exchange-bias effect in the LSMO/LNO interface of the BNTH_0.05_/LSMO/LNO film. It makes an obvious shift of the magnetization hysteresis to negative fields, yielding a non-zero value of ME voltage coefficient under DC bias magnetic field *H*_bias_ = 0 ^62^. Furthermore, the clamping effect originated from the LNO layer might be the other primary reason. The lattice constants of LNO and LSMO are 0.384 and 0.387 nm, respectively, which makes the composite film suffer a clamping effect, and leads to a compressive strain field. The strain field may cause the occurrence of the self-bias ME effect^63^. It is well known that most ferromagnetic-ferroelectric composite materials exhibit very weak ME response at near zero bias field (*H*_bias_ = 0 Oe). As a result, the requirement of additional large *H*_bias_ would be problematic for the application of ferromagnetic-ferroelectric composite materials in devices. The discovery of self-biased ME effect in the BNTH_0.05_/LSMO/LNO film is encouraging for exploring its potential applications such as self-biased magnetic field sensor^65^. Figure 11 also shows the *α*_E_ values response in the same frequency range at *H*_bias_ = 200 Oe, and the same trend that the *α*_E_ value gradually increased from zero with the increase of the AC frequency was observed. Compared with the *α*_E_ values at *H*_bias_ = 0 Oe, the corresponding values at *H*_bias_ = 200 Oe have only a small increase. The highest *α*_E_ value of 20 V/cm·Oe can be eventually obtained at the AC magnetic field frequency of 100 kHz. It is comparable to the highest values obtained in the most ferromagnetic-ferroelectric composite films^56, 60, 66^. Furthermore, it is necessary to add additional remarks that the trend that the *α*_E_ value increases with the increasing of the AC frequency was also observed in the NiFe_2_O_4_- Pb(Zr,Ti)O_3_ magnetoelectric composite ceramic by Nan *et al*.^67^, CoFe_2_O_4_-Pb(Zr,Ti)O_3_ magnetoelectric composite film by Wan *et al*.^55^ and BaTiO_3_/LSMO magnetoelectric bilayer film by Li *et al*.^53^. According to theory models, the dielectric constant and the capacitivity have something to do with the ME effect^68–72^. According to the equation in ref. 67, the relationship between *α*_E_ and dielectric constant can be expressed as follow:

3 $${\alpha }_{E}=\frac{Q}{{\varepsilon }_{r}{\varepsilon }_{0}SdH}=\frac{Q}{{\varepsilon }_{0}SdH}\,\frac{1}{{\varepsilon }_{r}}$$

where *Q* is the charge generated from the samples which is collected by a charge amplifier, *S* the area of the sample, d*H* the Ac magnetic field, and *ε*_0_ is the dielectric constant at vacuum, equal to 8.85 × 10^−12^ F/m. According to the Eq. (3), *α*_E_ has an inverse ratio to the dielectric constant (ɛ_r_). The dielectric constant of BNTH_0.05_/LSMO/LNO film slowly decreases with the increase of the frequency until up to 100 kHz (Fig. 9). This fact can well explain that *α*_E_ increased with the frequency increasing, and no any saturation occurred in the frequency range from 0 kHz to 100 kHz (Fig. 11). Although the magnetic frequency of 100 kHz is the ultimate range of our instrument (Super-ME, Quantum Design China), the change of *α*_E_ with the frequency above 100 kHz could also be deduced according to the dielectric constant in dependent of the frequency. Because there was an intense drop in the dielectric constant (ε_r_) in the frequency range from 100 kHz to 1000 kHz, *α*_E_ would likely to increase slowly at first and then increase sharply at a certain frequency. It is well known that the dielectric constant (ε_r_) of ferroelectric materials would generally become stable at a very high frequency. So it could be speculated that the *α*_E_ might attain the saturation value at a certain frequency above 1000 KHz. In summary, the *c*-axis oriented BNTH_0.05_/LSMO/LNO film exhibited an excellent ME effect. The remarkable ME coefficient performance might be attributed to the high *c*-axis orientation and the good microstructure of the BNTH_0.05_/LSMO/LNO film, the larger magnetostriction of LSMO ferromagnetic phase and the excellent ferroelectric properties of BNTH_0.05_ phase.

## Conclusion

The (00 *l*)-oriented LNO buffered layer, LSMO ferromagnetic layer and BNTH_x_ ferroelectric layer were successively fabricated onto the (001) Si substrate via all CSD method. The LNO layer could be used as the seed layer to control the crystalline structure and the preferential orientation of the overlying LSMO and BNTH_x_ layers. As a result, the oriented BNTH_x_/LSMO ferromagnetic-ferroelectric composite film with a 2–2 type structure was integrated on Si substrate. The Nd^3+^/Hf^4+^ co-substitution can really decrease the leakage current and improve ferroelectric properties of BIT film. It is attributed to the A-site substitution by Nd^3+^ that could enhance the stability of perovskite-like structure and reduce the oxygen vacancy concentration in BIT, and the B-site substitution by Hf^4+^ that could induce the distortion of oxygen octahedra and the decrease of the space charge density. For BNTH_x_ films, the BNTH_0.05_ film has the lowest leakage current density of 2.5 × 10^−7^ A/cm^2^ at 200 kV/cm, and the highest *P*r of 27.3 μC/cm^2^. The BNTH_0.05_/LSMO/LNO film exhibits the excellent ME effect, and its ME voltage coefficient value, *α*_E_, at the near zero *H*_bias_ magnetic field is very large, but its variation with *H*_bias_ is modest. In addition, the ME sensitivity of the composite film strongly depended on the frequency of *H*_ac_ even in the absence of *H*_bias_, suggesting the existence of self-biased magnetoelectric effect in the BNTH_0.05_/LSMO/LNO film. This contribution certifies that it is feasible to fabricate the *c*-axis oriented ferromagnetic-ferroelectric composite films including a bismuth-layered perovskite ferroelectric phase on Si substrates, and integrate lead-free ferromagnetic-ferroelectric composite film materials with other materials in silicon based devices.

## Methods

### Preparation

All metallic salts and organic reagents were purchased from Sigma-Aldrich and used as the starting materials without any further purification. The preparation of LNO, LSMO and BNTH_x_ solutions and its dip-coating processes for the gel films were performed in a home-made glove box where the relative humidity was controlled below 30%, and the temperature was set at 25 °C.

The LNO film was deposited on the (001) Si substrate by the CSD method as follow. Firstly, La(NO_3_)_3_·6H_2_O and Ni(CH_3_COO)_2_·4H_2_O was dissolved in a methanol (MeOH) solvent. Subsequently, acetyl acetone (AcAc) as a chelating agent was added. The molar ration of La(NO_3_)_3_·6H_2_O:Ni(CH_3_COO)_2_·4H_2_O:MeOH:AcAc is 1:1:125:1. After the mixture was continuously stirred for 24 h, a transparent and green colored LNO solution was obtained. The LNO gel film was prepared on the Si substrate by the dip-coating of the as-prepared LNO solution with a drawing rate of 0.8 mm/s. To prepare highly oriented LNO films, the dip-coated films were successively dried at 150 °C for 10 min, then heated up to 750 °C at a heating rate of 40 °C/s, and finally annealed for 10 min using a rapid thermal annealing (RTA) furnace in air atmosphere. To increase the conductivity of the LNO films, the dip-coating, drying and annealing processes were repeated for 8 times. Finally, the LNO films were re-annealed for the crystallization in a tube furnace with an oxygen flux of 100 mL/min. The resistivity of the LNO film measured by a four-probe tester was about 9.35 × 10^−4^ Ω·cm.

Subsequently, the LSMO layer was deposited onto the as-prepared LNO film by the CSD method. The precursor LSMO solution was prepared by dissolving La(NO_3_)_3_·6H_2_O, Sr(CH_3_COO_)2_·0.5H_2_O and Mn(CH_3_COO)_2_·4H_2_O in the mixture of MeOH and AcAc, and aging for 24 h. The molar ration of La(NO_3_)_3_·6H_2_O:Sr(CH_3_COO)_2_·0.5H_2_O:Mn(CH_3_COO)_2_·4H_2_O:MeOH:AcAc is 0.67:0.33:1:125:1. The drawing rate to cast a LSMO gel film was about 0.8 mm/s. Next, the coated LSMO gel films were pre-annealed in air at 350 °C for 10 min, then heated up to 750 °C at a heating rate of 40 °C/s, and finally annealed for 10 min using a RTA furnace. After dip-coating, drying, and annealing processes were repeated 5 times, the films were continuously crystallized in air at 750 °C for 1 h.

Thin layers of BNT, BNTH_0.025_, BNTH_0.05_, BNTH_0.1_ and BNTH_0.15_ were deposited onto the LSMO/LNO films using the dip-coating/annealing cycles. To prepare BNTH_x_ (x = 0, 0.025, 0.05, 0.10 and 0.15) solutions, appropriate Bi(NO_3_)_3_·5H_2_O, Nd(NO_3_)_3_·5H_2_O and HfCl_4_ were dissolved in a 2-methoxyethanol (MOE) solution and then stirred to clarify it. The molar ration of Bi(NO_3_)_3_·5H_2_O:Nd(NO_3_)_3_·5H_2_O:HfCl_4_:MOE is 3.465:0.85:x:137 (x = 0, 0.025, 0.05, 0.10 and 0.15). A 10% excess of Bi(NO_3_)_3_·5H_2_O was used to compensate for the loss of Bi occurring during the annealing process. Meanwhile, an amount of Ti(OC_4_H_9_)_4_ was dropped into the mixture of AcAc and MOE, and stirred at room temperature for 0.5 h. The molar ration of Ti(OC_4_H_9_)_4_:AcAc:MOE is 1:3.4:48. Next, two solutions were mixed in a molar ration of Bi: La:Ti:Hf = 3.465:0.85:(3−x):x (x = 0, 0.025, 0.05, 0.10 and 0.15), and stirred at room temperature for 0.5 h. Subsequently, an amount of MOE was added to keep 0.7 mol/L of the total metal ions concentration. A lactate stabilizer and an acetic anhydride dehydrating agent, respectively accounted for 1% of the total solution volume, was dropped and stirred at room temperature for 12 h. Finally, transparent light-yellow BNTH_x_ solutions were prepared. The BNTH_x_ gel layers were cast onto the LSMO/LNO films by the dip-coating at the drawing rate of 0.5 mm/s. After that, the dip-coated film was pre-annealed in air at 200 °C for 5 min, then heated up to 730 °C with a heating rate of 40 °C/min and continuously heated for 10 min in a RTA furnace to remove any organic residuals. After the dip-coating, drying and annealing procedure were repeated for 8 times, the entire films were re-annealed at 730 °C for 60 min in air atmosphere to improve its crystallization. Furthermore, In order to investigate the effect of the substitution on the electric properties of BNTH_x_ films, a pure BIT film was prepared using the same approach without any addition of Nd(NO_3_)_3_·5H_2_O and HfCl_4_. It was necessary to add that BNTH_x_ powders were prepared from their corresponding solutions by being dried at 80 °C in a drying box, then heated up to 730 °C at a heating rate of 15 °C/min, and continuously annealed for 3 h in a RTA furnace.

### Characterization

The crystal structure and crystalline orientation of all films were characterized by low-angle and theta-2 theta X-ray diffraction (XRD, Shimadzu, XRD-7000, Cu Kα radiation, λ = 1.5406 Å) with the sampling pitch of 0.02°, respectively. The low-angle XRD analysis was performed in reflection geometry by fixing 1° between the incident X-ray beam and the film surface, and rotating the detector angle. Raman spectra of BNTHx powders were carried out at room temperature using Renishaw inVia plus equipped with an argon ion laser at 514.5 nm. The chemical composition of BNTH_0.05_ film was identified by X-ray photoelectron spectroscopy (XPS, ESCALAB-250Xi) with Al Kα (1486.71 eV) line at the power of 150 W (10 mA, 15 kV). The surface morphologies of the BNTH_x_ films were characterized by field emission scanning electron microscopy (FE-SEM, JSM-6700F, JEOL). The BNTH_0.05_/LSMO/LNO/Si specimen was prepared by conventional grinding and polishing, and then examined using high resolution JEM-3010 TEM equipment with a lattice resolution of 0.14 nm. For ferroelectric and ME coupling measurements, Pt top electrodes were deposited onto the BNTH_x_ layers by the direct current sputtering through a shadow mask. The ferroelectric and leakage behaviors of the composite films were characterized using a ferroelectric tester (TF-Analyzer 2000, aixACCT). The magnetic hysteresis loop of the BNTH_0.05_/LSMO/LNO film was measured using a vibrating sample magnetometer (VSM) in a physical property measurement system (Versalab, Quantum Design) with an error margin of ±0.5%. The ME effect analysis of the BNTH_0.05_/LSMO/LNO film was performed using a ME measuring device (Super-ME, Quantum Design China).
